# ABCE Proteins: From Molecules to Development

**DOI:** 10.3389/fpls.2018.01125

**Published:** 2018-08-03

**Authors:** Carla Navarro-Quiles, Eduardo Mateo-Bonmatí, José L. Micol

**Affiliations:** Instituto de Bioingeniería, Universidad Miguel Hernández, Elche, Spain

**Keywords:** ABCE, ribosome, translation, development, RLI

## Abstract

Most members of the large family of ATP-Binding Cassette (ABC) proteins function as membrane transporters. However, the most evolutionarily conserved group, the ABCE protein subfamily, comprises soluble proteins that were initially denoted RNase L inhibitor (RLI) proteins. ABCE proteins are present in all eukaryotes and archaea and are encoded by a single gene in most genomes, or by two genes in a few cases. Functional analysis of *ABCE* genes, primarily in *Saccharomyces cerevisiae*, has shown that ABCE proteins have essential functions as part of the translational apparatus. In this review, we summarize the current understanding of ABCE protein function in ribosome biogenesis and recycling, with a particular focus on their known and proposed developmental roles in different species. The ABCE proteins might represent another class of factors contributing to the role of the ribosome in gene expression regulation.

## ABC protein structure, function, and classification

The ATP-Binding Cassette (ABC) proteins, which are present in all living organisms, constitute one of the largest known protein families. Actually, in prokaryotes, the ABC genes constitute 1–3% of the genome (Tomii and Kanehisa, 1998). The *Saccharomyces cerevisiae* and human genomes encode 30 and 48 ABC proteins, respectively (Dean et al., 2001; Paumi et al., 2009; Vasiliou et al., 2009). By contrast, in plants like *Arabidopsis thaliana*, there are more than 100 genes encoding ABC proteins (Verrier et al., 2008). Such multiplication and functional diversification of ABC proteins is consistent with the sessile nature of plants and their adaptation to changing terrestrial environments, as well as with the history of whole-genome duplications in plant evolution (Hwang et al., 2016).

Most ABC proteins transport solutes across cell membranes. These solutes, referred to as allocrites (Holland and Blight, 1999), range from small inorganic and organic molecules to large organic compounds. ABC transporters contain two transmembrane domains (TMDs) and two nucleotide-binding domains (NBDs), otherwise known as ATP-binding cassettes, which are hallmarks of the ABC family. TMDs and NBDs can be contained together in a unique full-sized protein, as commonly found in eukaryotes, or can be separated into individual peptides (subunits), as observed in prokaryotes. ABC proteins can also occur as homo- or heterodimers formed by half-sized proteins, which contain one TMD and one NBD or consist only of fused NBDs (Hyde et al., 1990). TMDs are responsible for allocrite specificity and have polyphyletic origins. TMDs belonging to a specific transporter subtype display similar membrane topologies among distant species. Each TMD typically comprises 6–10 α-helices that span the cell membrane, thus generating a pore that is accessible from the cytoplasm or from the extracellular space (Wang et al., 2009; Zheng et al., 2013).

In contrast to TMDs, NBDs are monophyletic with conserved sequences and structures (Higgins et al., 1986). The NBD regions that display the highest level of conservation are those that function specifically in ATP binding and hydrolysis, namely the Walker A and Walker B motifs; the LSGGQ signature or ABC motif; and the A-, D-, H-, and Q-loops, named after the conserved residues at their N- or C-termini (ter Beek et al., 2014). NBDs are arranged in a head-to-tail orientation, which allows them to bind two ATP molecules. These ATP molecules interact with motifs from both NBDs in a sandwich-like manner, bringing NBDs together in a closed conformation. ATP cleavage (hydrolysis) relaxes this conformation and drives the transport cycle by producing coupled conformational changes in the TMDs that recognize and translocate the allocrite across the membrane. Whether both NBDs stay in contact (continuous contact models) or totally separate (NBDs separation models) after ATP hydrolysis remains a matter of active debate. However, since there are several ABC transporter subtypes, it is reasonable to assume that more than one transport mechanism exists (Shi and Barna, 2015).

**Figure 1:**
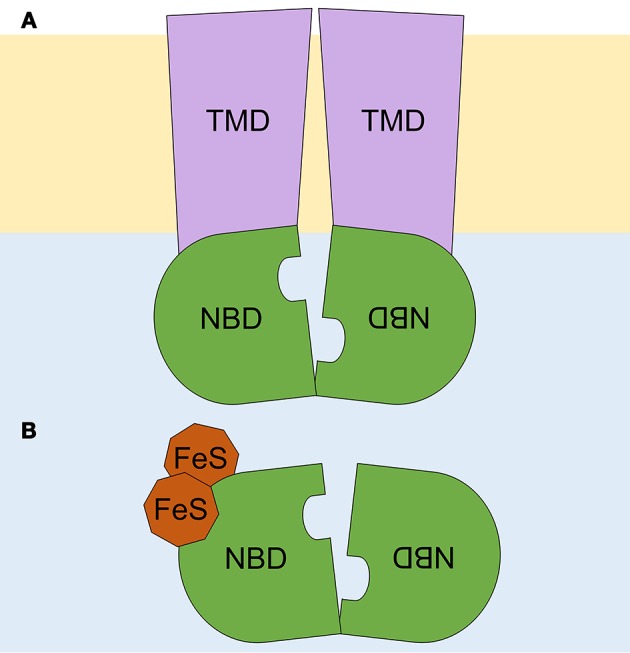
Schematic representation of ABC proteins. **(A)** An ABC transporter, and **(B)** an ABCE soluble protein. A lipid bilayer is depicted in pale orange, and the cytoplasm, in pale blue. Transmembrane domains (TMD) are shown in purple and nucleotide binding domains (NBD), arranged in a head-to-tail orientation, in green. The two diamagnetic [4Fe-4S]^2+^ clusters are depicted in brown. Adapted from Hopfner (2016).

To facilitate ABC protein recognition and comparison among different species, a standardized nomenclature was proposed for all eukaryotes (Dean and Annilo, 2005; Verrier et al., 2008; Paumi et al., 2009; Xie et al., 2012; Dermauw and Van Leeuwen, 2014). Mammalian ABC proteins were divided into seven subfamilies (ABCA to ABCG) based on NBD sequence similarity (Dean et al., 2001). Two additional subfamilies were later proposed for non-mammalian ABC proteins, specifically the ABCH subfamily, which is found in insects and osteichthyes (Dean and Annilo, 2005), and the ABCI subfamily, which is found exclusively in plants and contains prokaryotic-like ABC proteins, among others (Verrier et al., 2008). ABCA, ABCB, ABCC, ABCD, ABCG, and ABCH proteins function as transporters (Figure 1A; Hopfner, 2016).

By contrast, soluble ABC proteins belong to the ABCE and ABCF subfamilies, which lack TMDs but have the two NBDs in a single peptide chain (Figure 1B). ABCF proteins function in translation, as exemplified by eukaryotic elongation factor 3 (eEF3) (Andersen et al., 2006), or in chromosome segregation and DNA repair, as is the case for the Structural Maintenance of Chromosomes (SMC) proteins and the SMC-like protein Rad50 (Hirano, 2002). Here, we focus on the ABCE subfamily, which includes single-copy genes that, although initially thought to be specific to mammals (Bisbal et al., 1995), are found in both eukaryotes and archaea.

## The ABCE subfamily of ABC proteins

ABCE proteins display the highest level of conservation among the ABC subfamilies. For example, the yeast ABCE1/Rli1 shares 68 and 43% sequence identity with its human and archaeal (*Sulfolobus solfataricus*) orthologs, respectively (Kispal et al., 2005; Barthelme et al., 2007). The ABCE subfamily is represented in most genomes by a single-copy, essential *ABCE1* gene (Kerr, 2004). However, two *ABCE* paralogs, specifically *ABCE1/RLI1* and *ABCE2/RLI2*, exist in plants such as *Arabidopsis thaliana* and *Oryza sativa* (Sarmiento et al., 2006; Verrier et al., 2008), and in animals such as catfish (Liu et al., 2013) and the mosquitoes *Anopheles gambiae, Aedes aegypti*, and *Culex pipiens quinquefasciatus* (Lu et al., 2016).

Loss-of-function of *ABCE1* genes, either via null alleles or RNAi suppression, is associated with a lethal phenotype in all studied species, and hypomorphic *ABCE1* alleles result in slow-growth phenotypes (Amsterdam et al., 2004; Dong et al., 2004; Estévez et al., 2004; Zhao et al., 2004; Coelho et al., 2005a; Kispal et al., 2005; Chen et al., 2006; Barthelme et al., 2007; Broehan et al., 2013; Kougioumoutzi et al., 2013; Table 1). Conversely, in *Saccharomyces cerevisiae, RLI1* overexpression leading to accumulation of either the wild-type protein or mutated versions disrupted in conserved residues or lacking entire conserved domains caused a dominant negative effect on growth (Dong et al., 2004; Khoshnevis et al., 2010).

**Table 1:** Mutations affecting *ABCE* genes in different species.

**Organism**	**Gene name**	**Loss of function caused by**	**Phenotype**	**References**
*Drosophila melanogaster*	*pixie*	Strong hypomorphic alleles	Recessive lethal	Coelho et al., 2005a,b
		Weak hypomorphic alleles	Slow growth; disproportionate organ sizes; slender bristles; eye roughening	Coelho et al., 2005a,b
*Caenorhabditis elegans*	*abce-1*	RNAi	Slow growth; embryonic lethality	Kamath et al., 2003; Zhao et al., 2004
*Danio rerio*	*abce1*	Retroviral insertional allele	Small head and eyes; underdeveloped liver and gut; pericardial edema; lethal at 5 days post-fertilization	Amsterdam et al., 2004
*Xenopus laevis*	*abce1*	Antisense *ABCE1* morpholino oligonucleotides	Arrested growth at the gastrula stage	Chen et al., 2006
*Cardamine hirsuta*	*SIL3*; *ChRLI2*	Hypomorphic allele	Reduced growth; small and simple leaves; delayed leaf initiation; reduced auxin signaling; reduced cell proliferation; high rates of endoreplication	Kougioumoutzi et al., 2013
*Nicotiana benthamiana*	*RLIh*	Virus-induced gene silencing	Reduced growth; distorted leaves; whitened veins; reduced cell size and number	Petersen et al., 2004

Determination of the crystal structure of archaeal ABCE1 (aABCE1) proteins (Karcher et al., 2005, 2008; Barthelme et al., 2011) showed that these proteins contain four conserved domains: (1–2) the two NBDs that are present in all ABC proteins; (3) a hinge region proposed to facilitate NBD orientation and function as a pivot point in the tweezer-like power stroke of NBDs following ATP binding (Karcher et al., 2005); and (4) an iron-sulfur (FeS) binding domain with eight cysteine residues that coordinate two diamagnetic [4Fe-4S]^2+^ clusters present at the ABCE protein N-terminal region (Figure 1B; Barthelme et al., 2007; Karcher et al., 2008). The latter domain plays an essential role in ABCE protein function (Kispal et al., 2005; Yarunin et al., 2005; Barthelme et al., 2007, 2011; Alhebshi et al., 2012). The high-level conservation among all ABCE amino acid sequences, in particular within their four conserved domains, allows to deduce the structure of eukaryotic ABCE orthologs based on that of aABCE1 (Karcher et al., 2005, 2008). Moreover, cryoelectron microscopy showed that *Pyrococcus furiosus* aABCE1 and yeast Rli1 associate similarly with ribosomes (Becker et al., 2012; Preis et al., 2014).

**Figure 2:**
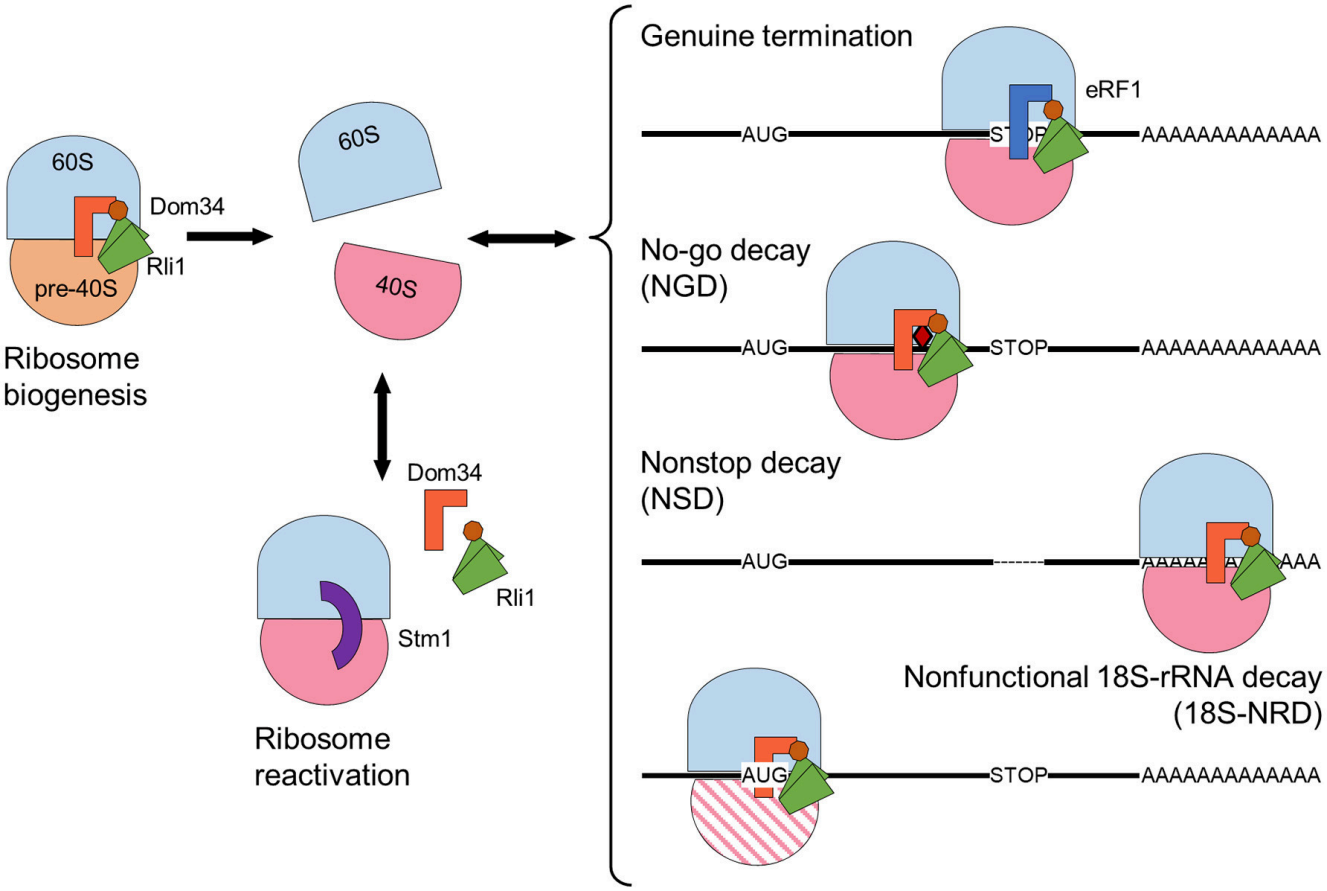
Rli1 ribosome-dissociation activity has essential functions in diverse cellular processes, including ribosome maturation and release of stalled ribosomes. Schematic representation of a maturing ribosome formed by 60S (blue) and pre-40S (orange) subunits. Mature 40S subunits are depicted in pink. A faulty 40S subunit is represented by pink parallel lines. Class I release factors Dom34 (orange) and eRF1 (dark blue) dissociate ribosomes together with Rli1, whose NBDs are represented in green, and the FeS domain in brown. The “clamping” factor Stm1 is depicted in purple. The no-go decay mRNA contains a secondary structure represented by a red rhombus. The non-stop mRNA lacks an in-frame stop codon, depicted by a dashed line. Arrows indicate ribosome association-dissociation flow.

The function of the yeast ABCE1 protein Rli1 has been well characterized. Furthermore, based on the sequence conservation, ABCE1 protein function is likely to be well conserved among different organisms. Yeast Rli1 triggers the dissociation of ribosomes during different processes related to translation, as described in further detail below (Figure 2). Additional roles have been proposed for ABCE proteins in higher eukaryotes. For example, ABCE1 participates in the assembly of immature HIV-1 capsids in mammals (Zimmerman et al., 2002; Dooher and Lingappa, 2004), and ABCE proteins act as endogenous suppressors of RNA silencing in plants and humans (Sarmiento et al., 2006; Kärblane et al., 2015).

## Yeast ABCE1/Rli1 functions in ribosome biogenesis and recycling

The *Saccharomyces cerevisiae* genome encodes 30 ABC proteins, including one member of the ABCE subfamily, Rli1. *RLI1* encodes a canonical ABCE protein and contains two NBDs arranged in a head-to-tail manner (thus allowing the binding of two ATP molecules), and a FeS domain (Barthelme et al., 2007). The *Drosophila melanogaster* Rli1 ortholog, Pixie, localizes exclusively in the cytoplasm (Coelho et al., 2005a), whereas yeast Rli1 localizes in the cytoplasm and in the nucleus (Dong et al., 2004; Kispal et al., 2005; Yarunin et al., 2005).

Yeast Rli1 associates with eukaryotic translation initiation factors, 40S ribosomal subunits, 80S ribosomes, and polysomes. Suppression of *Rli1* strongly reduces polysome size and abundance, as well as translation rates (Dong et al., 2004; Kispal et al., 2005; Yarunin et al., 2005; Shoemaker and Green, 2011). These observations suggested that Rli1 participates in translation initiation by promoting assembly of the preinitiation complex. Equivalent observations were made for human ABCE1 (Chen et al., 2006) and *Drosophila* Pixie (Andersen and Leevers, 2007). Additionally, ABCE proteins in *Trypanosoma brucei* (Estévez et al., 2004) and *Caenorhabditis elegans* (Zhao et al., 2004) were also implicated in translation. A role for yeast Rli1 and mammalian ABCE1 in translation initiation was confirmed following analysis of the 48S initiation and the post-splitting complexes (Heuer et al., 2017; Mancera-Martínez et al., 2017).

The role of Rli1 as a ribosome biogenesis factor is supported by the nuclear accumulation of 40S and 60S ribosome subunits when Rli1 function is compromised (Kispal et al., 2005; Yarunin et al., 2005). Nevertheless, the most well-known Rli1 function is that of ribosome disassembly (Figure 2). Indeed, Rli1 facilitates ribosomal subunit dissociation through its direct interaction with the class I release factor Supressor 45 (Sup45), otherwise known as eukaryotic release factor 1 (eRF1) or its paralog Duplication of Multilocus region 34 (Dom34). Such interaction was confirmed by affinity pull-down, coimmunoprecipitation, and yeast two-hybrid analyses (Khoshnevis et al., 2010; Shoemaker and Green, 2011).

### Yeast Rli1 participates in ribosome recycling

Translation termination occurs when the stop codon of an mRNA enters the A site of the associated ribosome, after which the tRNA-mimicking eRF1 recognizes the stop codon via codon-anticodon recognition using its conserved NIKS (Asn-Ile-Lys-Ser) motif and occupies the A site of the ribosome (Song et al., 2000). Following this, the GTP bound to the eRF1-linked class II release factor Sup35/eRF3 is hydrolyzed, thus dissociating the post-termination complex.

During stop codon recognition, eRF3 has been proposed to facilitate the interaction between eRF1 and the ribosome. This function is thought to resemble delivery of aminoacylated tRNA to the ribosome A site during peptide elongation, which is performed by the eRF3 paralog eEF1α (Inagaki et al., 2003; Salas-Marco and Bedwell, 2004; des Georges et al., 2014). In an alternative scenario, DEAD-box protein 5 (Dbp5), an RNA helicase that participates in mRNA export, recruits eRF3·GTP to the ribosome following eRF1 stop codon recognition (Gross et al., 2007). Regardless of the exact protein-protein interactions, eRF3 must break down GTP and leave the ribosome to allow Rli1 binding (Shoemaker and Green, 2011; Preis et al., 2014).

The kinetic analysis of an *in vitro* reconstituted yeast translation system demonstrated that Rli1 induces eRF1 ribosome accommodation in an ATP-independent manner. Ribosome accommodation allows eRF1 to catalyze peptidyl-tRNA hydrolysis through its conserved GGQ (Gly-Gly-Gln) motif, which releases the newly synthesized peptide. ATP hydrolysis driven by Rli1 promotes ribosome subunit dissociation, demonstrating that eukaryotic translation termination and ribosome recycling are combined within the same release factor-mediated process. Such combination contrasts with the separation of the two processes observed in bacteria (Shoemaker and Green, 2011). In this manner, the 60S subunit is disassembled from the 40S subunit, which is then released from the deacylated tRNA and mRNA molecules. During all this process of ribosome recycling, Rli1 remains bound to the 40S subunit and has been suggested to preclude 60S rejoining until a late-stage in the initiation complex (Heuer et al., 2017; Mancera-Martínez et al., 2017). In the case of archaea, it has been proposed that ribosome dissociation is caused by a conformational change following aABCE1-ribosome interaction, and that ATP hydrolysis is required to separate aABCE1 from the 30S subunit following ribosome dissociation (Barthelme et al., 2011; Kiosze-Becker et al., 2016).

Termination of translation can be inefficient. One of the known causes of inefficient translation termination is the continued association of ribosomes with defective mRNA molecules, which impairs translation and produces potentially deleterious peptides. To circumvent this, different mRNA surveillance pathways can degrade defective mRNA molecules and their translation products, and recycle the associated ribosomes. These mRNA surveillance pathways have been extensively reviewed (Franckenberg et al., 2012; Graille and Séraphin, 2012; Shoemaker and Green, 2012; Brandman and Hegde, 2016), so they are only briefly discussed here.

Each of the three primary mRNA surveillance mechanisms targets a different cause of ribosome stalling and Rli1 participates in all three mechanisms. In no-go decay (NGD), a physical obstruction slows down or stops ribosome progression on the mRNA molecule. Physical obstructions can include an inhibitory secondary structure, chemical damage, or a polybasic sequence within the nascent protein (Doma and Parker, 2006; Kuroha et al., 2010). Non-stop decay (NSD) occurs when the mRNA lacks a genuine stop codon, possibly due to truncation or premature polyadenylation of the mRNA molecule. In NSD, the ribosome continues translation until it encounters an in-frame stop codon on the 3′ UTR or comes to the poly(A) mRNA sequence (tail). Translation of the poly(A) tail generates a positively charged poly-lysine region that disturbs ribosome movement by interacting with its negatively charged translation tunnel (Frischmeyer et al., 2002; Ito-Harashima et al., 2007; Lu and Deutsch, 2008; Guydosh and Green, 2014). Lastly, non-functional 18S rRNA decay (18S-NRD) repairs errors in translation that are caused by dysfunctional ribosomes carrying an inactive or immature 40S subunit. 18S-NRD rapidly removes these faulty ribosomes that have initiated translation, but cannot produce an elongating peptide (LaRiviere et al., 2006; Soudet et al., 2010).

Each of these surveillance systems uses the same basic ribosome rescue machinery components. Ribosome rescue starts with recognition of the stalled ribosome by a ternary complex formed by Dom34 and Hsp70 subfamily B Suppressor 1 (Hbs1·GTP), which are paralogs of eRF1 and eRF3, respectively (Cole et al., 2009; Shoemaker et al., 2010; Tsuboi et al., 2012). Following this, Rli1 dissociates the ribosome into the 40S and 60S subunits via a similar mechanism as during the normal termination of translation. Dom34 lacks the conserved NIKS motif that is involved in stop codon recognition and the GGQ motif that catalyzes peptide release, which are characteristic features of eRF1 (Graille et al., 2008; Shoemaker et al., 2010).

The mRNA surveillance pathways appear to be conserved among all eukaryotes and archaea. For example, Pelota, the Dom34 paralog in *Drosophila melanogaster*, can restore NGD in Dom34-depleted yeast cells (Passos et al., 2009). Also, the human and fly Pelota-Hbs1 complex, together with ABCE1/Pixie, participates in NSD (Pisareva et al., 2011; Saito et al., 2013; Kashima et al., 2014). In archaea, the elongation factor aEF1α, an ortholog of eRF3, interacts with aRF1 during the normal termination of translation and aPelota during mRNA surveillance, resulting in ribosome dissociation via aABCE1 action (Saito et al., 2010; Barthelme et al., 2011; Becker et al., 2012).

### Rli1 is required for ribosome biogenesis and reactivation

Yeast ribosome biogenesis begins in the nucleolus where the 35S and 5S rDNAs are transcribed. Following this, pre-35S and pre-5S rRNAs are cotranscriptionally assembled with ribosomal proteins, ribosome biogenesis factors (RBFs), and small nucleolar ribonucleoproteins (snoRNPs) to form the 90S or small subunit (SSU) processome, which is the earliest ribosome precursor. Cleavage of the 35S pre-rRNA creates the pre-60S and pre-40S particles, and the maturation of these continues in the nucleoplasm and the cytoplasm (Gerhardy et al., 2014). Once in the cytoplasm, RBFs prevent premature translation initiation on immature pre-60S and pre-40S particles (Gartmann et al., 2010; Strunk et al., 2011; Greber et al., 2012).

Maturation of the pre-60S particle is complete when the pre-6S rRNA is processed to form 5.8S rRNA, all ribosomal proteins are assembled, and the last RBFs are released (Lo et al., 2010). The last step in 40S subunit maturation is performed in a translation-like cycle, whereby the initiation factor eIF5B links a pre-40S particle to a mature 60S subunit to form an empty 80S-like ribosome, which is necessary for 20S pre-rRNA cleavage into mature 18S rRNA. This process also serves as an additional checkpoint that, together with those performed during subunit maturation, ensures functionality of the ribosome (Strunk et al., 2012; Karbstein, 2013). Finally, Rli1, together with Dom34 and possibly Hbs1, dissociates the ribosomal subunits, which are then ready to enter the translation cycle (Shoemaker and Green, 2011; Strunk et al., 2012). Likewise, human Pelota and ABCE1 can dissociate empty ribosomes *in vitro* (Pisareva et al., 2011).

Protein synthesis is a cyclic process in which ribosomal subunits dissociate once they have processed an mRNA molecule and subsequently can reinitiate translation. However, in yeast cells subjected to stress conditions, some ribosomal subunits are complexed into inactive 80S ribosomes to reduce translation rates and increase the probability of surviving (Ashe et al., 2000; Uesono and Toh, 2002). In yeast, these inactive ribosomes are stabilized by the Suppressor of ToM1 (Stm1) factor (Balagopal and Parker, 2011; Ben-Shem et al., 2011; Van Dyke et al., 2013). Ribosomal inactivation is reversed once stress conditions are relieved and the ribosomal subunits can reenter the translation cycle. In glucose-starved yeast cells, dissociation of Stm1-bound ribosome requires the combined action of the Dom34-Hbs1 complex and Rli1. When yeast cells are grown in glucose-deficient media, translation rates rapidly decrease, associated with a decline in polysome levels and the accumulation of Stm1-inactivated 80S ribosomes. With the addition of glucose, translation rapidly resumes; however, Dom34 or Hbs1 loss-of-function prevents the recovery of translation, which causes a cessation in growth (Ashe et al., 2000; van den Elzen et al., 2014).

## The role of ABCE proteins in development

Yeast Rli1 is the best-characterized ABCE protein. In yeast and the unicellular protist *Trypanosoma brucei, ABCE1* loss-of-function arrests growth (Dong et al., 2004; Estévez et al., 2004). Similar observations have been made in multicellular eukaryotes, showing that in some species, *ABCE1* loss-of-function and overexpression both result in impaired growth (Table 1; Amsterdam et al., 2004; Estévez et al., 2004; Zhao et al., 2004; Coelho et al., 2005a; Chen et al., 2006; Kougioumoutzi et al., 2013). Consistent with the fundamental role of ABCE1 in ribosome biogenesis and recycling, *ABCE1* expression is detected in most tissues and developmental stages in all studied organisms (Du et al., 2003; Zhao et al., 2004; Maeda et al., 2005; Sarmiento et al., 2006; Kougioumoutzi et al., 2013).

**Figure 3:**
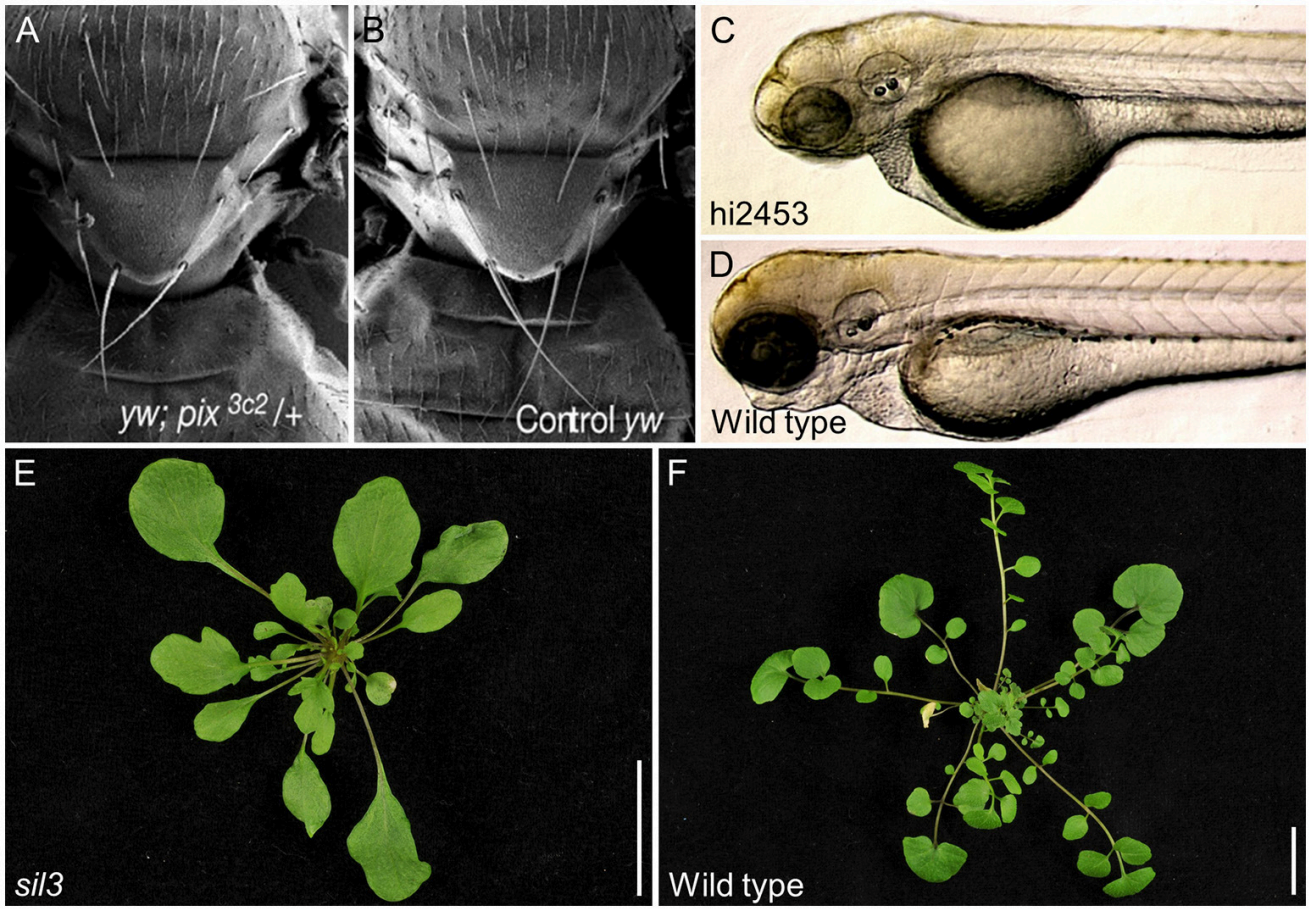
Developmental effects of *ABCE* gene dysfunction in different species. **(A)** *Drosophila melanogaster pixie* mutants display thoracic bristles that are slenderer and shorter than those of **(B**) the wild type. **(C)** An insertional allele of *Danio rerio ABCE1* reduces head size compared with **(D)** wild type and produces pericardial edema and lethality at 5 days post-fertilization. **(E)** A hypomorphic allele of *Cardamine hirsuta SIL3* triggers loss of the leaflets that characterize **(F)** wild type leaves. Pictures of **(A,B)**, as well as **(C,D)**, were taken at the same scale. **(E,F)** Scale bars indicate 2 cm. Adapted with permission of authors and journals from **(A,B)** Coelho et al. (2005a), **(C,D)** Amsterdam et al. (2004) Copyright 2004 National Academy of Sciences, and **(E,F)** Kougioumoutzi et al. (2013).

Many of the effects caused by *ABCE1* loss-of-function in eukaryotes have been revealed through genetic screens. For example, the *pixie* alleles were identified in a screen for ethyl methanesulfonate-induced dominant modifiers of a small-wing phenotype in *Drosophila melanogaster* (Coelho et al., 2005b). Null *pixie* alleles are recessive lethal, whereas hypomorphic alleles produce a severe delay in growth (Table 1). Nevertheless, the final body size of *pixie* mutants is comparable to that of the wild type. The *pixie* mutant phenotype resembles the *Minute* phenotype, which is associated with mutant alleles of genes encoding ribosomal proteins. Adult *pixie* flies display short thoracic bristles, occasional eye roughening, and an increased wing size relative to body size (Figures 3A,B). The increased wing size relative to body size is proposed to be due to extra cell divisions that act as a compensation mechanism triggered by high-level apoptosis observed in the wing imaginal discs during the development of *pixie* larvae (Coelho et al., 2005a).

ABCE proteins also function in vertebrate development. In a large screen for essential genes in embryo and early larval development in *Danio rerio*, mutations in *ABCE1* were found to cause lethality 5 days after fertilization. These *abce1* mutants exhibited underdeveloped liver/gut, pericardial edema, and small heads. Like the hypomorphic *pixie* alleles, the zebrafish *ABCE1* alleles also limit eye development (Table 1 and Figures 3C,D; Amsterdam et al., 2004). The *abce1* gene is also essential in *Caenorhabditis elegans* and *Xenopus laevis* as its suppression by RNAi or antisense morpholino oligonucleotides, respectively, arrests growth (Table 1; Kamath et al., 2003; Zhao et al., 2004; Chen et al., 2006). The ability of the *ABCE1*-suppressed zebrafish and *Xenopus laevis* embryos to develop up to a certain point has been suggested to be due to the ABCE1 maternal supply to the egg. The growth arrest would therefore occur after depletion of the maternal supply (Amsterdam et al., 2004; Chen et al., 2006).

*ABCE1* genes also have essential functions in plants. In *Cardamine hirsuta, SIMPLE LEAF3* (*SIL3*, also named *ChRLI2*) plays a role in leaf complexity. While wild-type *Cardamine hirsuta* plants display compound leaves, which are divided into leaflets, homozygous plants carrying the putatively hypomorphic *sil3* allele show a substantial decrease in leaflet but not leaf number (Table 1 and Figures 3E,F; Kougioumoutzi et al., 2013). The *sil3* mutant has a reduced growth and its phenotype suggests alterations in auxin homeostasis at the whole-organism level. Further proof of perturbed auxin homeostasis was found by analyzing *sil3* leaves, which are small and exhibit aberrant venation patterns, as usually observed in mutants affected in auxin signaling. In addition, the lack of leaflets in *sil3* plants was explained by reduced cell proliferation on the regions where leaflets were expected to emerge. Nevertheless, leaf and leaflet initiation are controlled by the same general mechanisms that consist in the accumulation of auxin by its polarized flow through the PIN-FORMED1 (PIN1) transporters in the regions where leaf initiation occurs. Auxin accumulation then triggers leaf or leaflet initiation (Scarpella et al., 2010). Although the study of the *sil3* mutant is consistent with auxin homeostasis and signaling being sensitive to perturbation of ribosomal activity, the *sil3* mutant is interesting because leaflet number, but not leaf number, is reduced. To explain this observation, it has been suggested that the high energy demand from cells proliferating during leaflet development could not be satisfied in *sil3* plants due to suboptimal ribosome function (Kougioumoutzi et al., 2013). The two Arabidopsis *ABCE* genes have not been studied at a developmental level.

In addition, virus-induced gene silencing (VIGS) has been used to suppress expression of *RLIh* gene(s) in *Nicotiana benthamiana*, in which the number of *ABCE* paralogs remains to be established. For VIGS, 4-week-old plants were infected with potato virus X (PVX) or pea early browning virus (PEBV) vectors carrying partial *RLIh* cDNAs. *RLIh* silencing caused vein whitening, leaf distortion, and delayed growth, with silenced plants reaching only half of the height of controls, due to a reduction in cell size and number in shoot internodes (Table 1; Petersen et al., 2004). Again, these observations corroborate the important role of ABCE proteins in whole-organism development. Whether these developmental defects are due to the disruption of the ABCE function as a ribosome-dissociating factor (Franckenberg et al., 2012), an endogenous suppressor of RNA silencing (Sarmiento et al., 2006; Kärblane et al., 2015) or both, remains to be clarified.

## Concluding remarks

In this review, we have discussed the essential ribosome-dissociation activity of ABCE proteins, which is required for ribosome biogenesis and recycling. Furthermore, we have described the general growth defects associated with compromised ABCE protein function in all studied organisms. Most of these growth defects can be attributed to defective mRNA translation, which reduces protein levels and in turn prevents cells from generating the energy required for normal growth and/or proliferation. However, not all *abce* mutant phenotypes can be explained by a general depletion of cellular energy. As is the case for mutants affected in translation in several species, some phenotypes appear to be associated with compromised regulatory networks that remain uncharacterized. For instance, it is striking the fact that the *Cardamine hirsuta sil3* mutant shows a reduction in leaflet but not leaf number, given that both processes share common pathways. Future work is needed to clarify these and other questions and to determine whether there are specific factors responsible for the differential requirements for ABCEs during development. Additional studies will also determine how ABCE dysfunction causes specific developmental aberrations in all studied organisms, as has been previously described for other proteins involved in ribosome biogenesis or function. In addition, the presence of more than one ABCE subfamily member in some genomes remains to be explained. Some plants and insects have two *ABCE* genes while other plants or *Drosophila melanogaster* have a single *ABCE* gene. The presence of two *ABCE* genes might be interpreted as an in-progress pseudogenization or a developing functional redundancy.

The ribosome has been proposed to represent a layer of post-transcriptional regulation of gene expression. Under the so-called filter hypothesis, the ribosome is viewed as a machine able to selectively influence or filter the translation of various mRNAs (Mauro and Edelman, 2002). There is increasing evidence of the existence of specialized ribosomes in different cell types, which are heterogeneous in either ribosomal protein composition, in their interactions with ribosome-associated factors, or both (Xue and Barna, 2012; Shi and Barna, 2015). These specialized ribosomes would differentially translate different mRNAs or mRNA groups. It follows from these assumptions that mutation of genes encoding discrete ribosomal proteins or ribosome-associated factors would render tissue- or organ-specific phenotypes. The ABCE proteins of *Drosophila melanogaster, Caenorhabditis elegans*, and *Cardamine hirsuta* might represent one such factors.

## Author contributions

JLM designed the review. JLM, EM-B and CN-Q analyzed all literature data, prepared Figures 1–3, and Table 1 and wrote the manuscript. All authors have accepted the final version of the manuscript and agreed to be accountable for all aspects of the work.

### Conflict of interest statement

The authors declare that the research was conducted in the absence of any commercial or financial relationships that could be construed as a potential conflict of interest.
